# Supplementary material for: Uptake of intrauterine contraception after medical management of first trimester incomplete abortion: A cross-sectional study in central Uganda
Source: PLoS One. 2025 Oct 7;20(10):e0310936. doi: 10.1371/journal.pone.0310936 (PMC12503278; doi:10.1371/journal.pone.0310936)
Supplement: S1 Appendix — (DOCX) [file pone.0310936.s001.docx]

**Appendix 1: Descriptive characteristics of five selected health facilities in central Uganda**

| **Health Unit** | **Location** | **Bed capacity** | **No. of healthcare providers** | **Duration of work** | **Cumulative 1^st^ trimester Abortion load in the past 3 months** | **Setting** | **Service delivered** |
| --- | --- | --- | --- | --- | --- | --- | --- |
| Kawempe National Referral Hospital | Kampala | 900 | 500 | 24 hours a day, 7 days a week. | 484 | Urban | Teaching hospital.  Offers free emergency gynaecological and obstetric services. |
| Mityana General Hospital | Mityana | 100 | 116 | 24 hours a day, 7 days a week. | 100 | Peri-urban | Offers comprehensive maternal health services including PAC. |
| Kiganda Health Centre IV | Kassanda | 15 | 50 | 24 hours a day, 7 days a week | 38 | Rural | Offers both in and outpatient services including family planning. |
| Kayunga Regional Referral Hospital | Kayunga | 200 | 179 | 24 hours a day, 7 days a week | 212 | Peri-urban | Offers specialist maternal services including PAC. |
| Bukuya Health centre III | Kassanda | 20 | 30 | 24 hours a day, 7 days a week | 60 | Rural | Offers maternal services including PAC, family planning services. |
