# Supplementary material for: Uptake of intrauterine contraception after medical management of first trimester incomplete abortion: A cross-sectional study in central Uganda
Source: PLoS One. 2025 Oct 7;20(10):e0310936. doi: 10.1371/journal.pone.0310936 (PMC12503278; doi:10.1371/journal.pone.0310936)
Supplement: Attachment — Submitted filename: renamed_97f81.pdf [file pone.0310936.s007.pdf]

Reviewers comments

1. Line 98….. two percent among……. and 15% among …….,,

I suggest the writer change two percent to 2% or change the 15% to fifteen percent to ensure uniformity. The same must be done for line 311 and all others.

2. Line 141 and 148 are contradictory. In 141, the writers say ultrasound was one of the modalities for dating the pregnancy. But in line 148 they say they only used ultrasound when team suspected incomplete miscarriage or device expulsion. I think they must reconcile the two statements.

3. Lines 150-156 how did the writers obtain consent from the minors who participated in the study(less 18 years). Did they involve their parents/guardians?

4. Lines 175- 181. should be revised . It's difficult to understand what the writers are saying.

I think the sentence should read, …At the post abortion clinic, potential participants with incomplete abortion were given comprehensive information on either ……..

5. Lines 178-181 " In Uganda , nurses-midwives are permitted to perform abortion….." I do not see the relevance of this statement under data collection. It may be appropriate to insert it under introduction.

1. Lines 297-298 should be captured on the table of results

The writers may address the issues raised above.
