# Supplementary material for: Uptake of intrauterine contraception after medical management of first trimester incomplete abortion: A cross-sectional study in central Uganda
Source: PLoS One. 2025 Oct 7;20(10):e0310936. doi: 10.1371/journal.pone.0310936 (PMC12503278; doi:10.1371/journal.pone.0310936)
Supplement: Attachment — Submitted filename: Peer review plos one_250124_124014.docx [file pone.0310936.s002.docx]

Most of unwanted pregnacies end up in induced abortion ......68

Unnecessary references	5......70

Statement with ref 7, check and revise......73

Using 7 ref for a statement seem overboard, 2-3 max would be advised....90,91

The content of description of the study setting is too much. Summary and make it more concise especially the management protocol of miscarriage.

Appendix should come after the whole work, not within the main text.

Participants recruitment description also too expansive.

Waiting for 4 week post management of miscarriage before recruitment pose risk of another preg, loss to follow up and apathy to use...why not preferably order the contraceptive before

discharge.....177-180

Could have been better to use iud uptake prevalence from study in Uganda and not south Asia or another country. .....187

Why using 2 different sample size calculation. Better to stick with the one gotten from Makenzius which emphasised post abortion iud update of iucd.

The methodology is too voluminous...all the sections

Statement in line	363-364...reference

Revise the statement in line 391.

....in our study as similar finding was report by Kayi et al in Ghana [ 56]

More point high uptake among married than unmarried... young, unmarried low use could be healthcare providers influence as most unmarried, single have multiple sexual partners which could heighten the risk of PID with iucd use.

Therefore discourage this age group from choosing it.

Reference

Ref 1.......496...remove the h

Revise the references with emphasis on placement of punctuation.

Good article.

Too voluminous. Word count should be reduced greatly.

Tables may be repeated from result section and come with appendix after main text.

The analysis is not appropriate. The test of association should have been done using chi square. If you after that decide to determine the test of the association, then

might proceed with logistic regression. You just did logistic regression and I don't feel so comfortable with that. Kindly revise.
