# Supplementary material for: Uptake of intrauterine contraception after medical management of first trimester incomplete abortion: A cross-sectional study in central Uganda
Source: PLoS One. 2025 Oct 7;20(10):e0310936. doi: 10.1371/journal.pone.0310936 (PMC12503278; doi:10.1371/journal.pone.0310936)
Supplement: Attachment — Submitted filename: Response to reviewer comments_uptake final.docx [file pone.0310936.s004.docx]

P.O. Box 7072, Kampala, Uganda

Phone: +256-414-534361

e-mail: [obsgyn@chs.mak.ac.ug](mailto:obsgyn@chs.mak.ac.ug)

## COLLEGE OF HEALTH SCIENCES

## SCHOOL OF MEDICINE

## DEPARTMENT OF OBSTETRICS AND GYNAECOLOGY

13^th^ March 2025

RESPONSE TO REVIEWS COMMENTS:

***Object:* PONE-D-24-39454** ***“UPTAKE OF INTRAUTERINE CONTRACEPTION AFTER MEDICAL MANAGEMENT OF FIRST TRIMESTER INCOMPLETE ABORTION: A CROSS-SECTIONAL STUDY IN CENTRAL UGANDA”***

With great pleasure, I’m thankful for your comments towards our manuscript. In response to the reviewers’ comments sent to us on 13^th^ March 2025, we have revised the manuscript accordingly.

| **Comment** | **Response to Comment** | **Page No. and Line** |
| --- | --- | --- |
| Dear Dr. Kayiga,  We've checked your submission and before we can proceed, we need you to address the following issues:  1. However, we note that there is identifying data in the Supporting Information file 'Use of IUDs updated..dta'. Prior to sharing human research participant data, authors should consult with an ethics committee to ensure data are shared in accordance with participant consent and all applicable local laws. Data sharing should never compromise participant privacy. It is therefore not appropriate to publicly share personally identifiable data on human research participants.  The following are examples of data that should not be shared: - Name, initials, physical address - Ages more specific than whole numbers - Internet protocol (IP) address - Specific dates (birth dates, death dates, examination dates, etc.) - Contact information such as phone number or email address - Location data - ID numbers that seem specific (long numbers, include initials, titled “Hospital ID”) rather than random (small numbers in numerical order)  Data that are not directly identifying may also be inappropriate to share, as in combination they can become identifying. For example, data collected from a small group of participants, vulnerable populations, or private groups should not be shared if they involve indirect identifiers (such as sex, ethnicity, location, etc.) that may risk the identification of study participants.  Additional guidance on preparing raw data for publication can be found in our Data Policy (<https://journals.plos.org/plosone/s/data-availability#loc-human-research-participant-data-and-other-sensitive-data>) and in the following article: <http://www.bmj.com/content/340/bmj.c181.long>.  Please remove or anonymize all personal information (Study Identification Number, Date of Recruitment, Religion, Register Unit Number, etc.), ensure that the data shared are in accordance with participant consent, and re-upload a fully anonymized data set. We also request that the dataset be shared in Excel (.xlsx or .csv) format. Please note that spreadsheet columns with personal information must be removed and not hidden as all hidden columns will appear in the published file.  In addition, please upload the following files as 'Other' files instead of Supporting Information files:  Full registration PhD-3.pdf Institutional clearance for all Health Facilities FP.pdf UNCST approval letter Family planning.pdf Revised consent forms 2.pdf Approved protocol-2.pdf Approval letter SOMREC.pdf Approval letter.pdf  We've returned your manuscript to your account. Please resolve these issues and resubmit your manuscript within 21 days. If you need more time, please email the journal office at [plosone@plos.org](mailto:plosone@plos.org). We are happy to grant extensions of up to one month past this due date. If we do not hear from you within 21 days, we will withdraw your manuscript.  t  . | Identifying data has been removed from the shared stata dataset as advised by the editor.  .  Identifying data has been removed from the shared stata dataset as advised by the editor. An Excel format has been used as advised.  Spreadsheet columns with personal information have been removed as advised.  Other files have been used for the mentioned files as advised.  Thanks so much for quick response otherwise. |  |

Yours truly,

**Dr Herbert Kayiga**

Lecturer/Corresponding Author,

Department of Obstetrics and Gynaecology

Makerere University College of Health Sciences

+256777855063, [hkayiga@gmail.com](mailto:hkayiga@gmail.com)
