# Supplementary material for: Uptake of intrauterine contraception after medical management of first trimester incomplete abortion: A cross-sectional study in central Uganda
Source: PLoS One. 2025 Oct 7;20(10):e0310936. doi: 10.1371/journal.pone.0310936 (PMC12503278; doi:10.1371/journal.pone.0310936)
Supplement: Attachment — Submitted filename: Response to reviewer comments Uptake manuscript final.docx [file pone.0310936.s006.docx]

P.O. Box 7072, Kampala, Uganda

Phone: +256-414-534361

e-mail: [obsgyn@chs.mak.ac.ug](mailto:obsgyn@chs.mak.ac.ug)

## COLLEGE OF HEALTH SCIENCES

## SCHOOL OF MEDICINE

## DEPARTMENT OF OBSTETRICS AND GYNAECOLOGY

13^th^ June 2025

RESPONSE TO REVIEWS COMMENTS:

***Object:* PONE-D-24-39454R2** ***“UPTAKE OF INTRAUTERINE CONTRACEPTION AFTER MEDICAL MANAGEMENT OF FIRST TRIMESTER INCOMPLETE ABORTION: A CROSS-SECTIONAL STUDY IN CENTRAL UGANDA”***

With great pleasure, I’m thankful for your comments towards our manuscript. In response to the reviewers’ comments sent to us on 10^th^ June 2025, we have revised the manuscript accordingly.

| **Comment** | **Response to Comment** | **Page No. and Line** |
| --- | --- | --- |
| JOURNAL REQUIREMENTS:  Please review your reference list to ensure that it is complete and correct. If you have cited papers that have been retracted, please include the rationale for doing so in the manuscript text, or remove these references and replace them with relevant current references. Any changes to the reference list should be mentioned in the rebuttal letter that accompanies your revised manuscript. If you need to cite a retracted article, indicate the article’s retracted status in the References list and also include a citation and full reference for the retraction notice.  **Additional Editor Comments:**  line 134; Resmove "see" before "appendix"  Line 282 add "P-value = 0.57"  line 284. Insert a reference for this sentence/assertion  line 296 . Remove "see" from "see Fig". Do the same for all tables and fig Table 2. Please include the Exact P-values of the adjusted PR in the Table.  Line 526 . Change I'm to "I am"  Please review all the references and ensure they conform with Vancouver referencing styles. Many of the references are not in line with Vancouver.   [Note: HTML markup is below. Please do not edit.]  Reviewers' comments:  Reviewer's Responses to Questions  **Comments to the Author**  1. If the authors have adequately addressed your comments raised in a previous round of review and you feel that this manuscript is now acceptable for publication, you may indicate that here to bypass the “Comments to the Author” section, enter your conflict of interest statement in the “Confidential to Editor” section, and submit your "Accept" recommendation.  Reviewer #2: (No Response)  Reviewer #4: All comments have been addressed  2. Is the manuscript technically sound, and do the data support the conclusions?  The manuscript must describe a technically sound piece of scientific research with data that supports the conclusions. Experiments must have been conducted rigorously, with appropriate controls, replication, and sample sizes. The conclusions must be drawn appropriately based on the data presented.  Reviewer #2: Partly  Reviewer #4: Yes  3. Has the statistical analysis been performed appropriately and rigorously?  Reviewer #2: No  Reviewer #4: Yes  4. Have the authors made all data underlying the findings in their manuscript fully available?  The [PLOS Data policy](http://track.editorialmanager.com/CL0/http:%2F%2Fwww.plosone.org%2Fstatic%2Fpolicies.action%23sharing/1/010f019757c10e09-9fd80757-f73f-4323-903e-2b216d7ff864-000000/rhbkTOBJzJ-RsdjDS5CWu8xmhy4ERP-7xks_nstQtG4=214) requires authors to make all data underlying the findings described in their manuscript fully available without restriction, with rare exception (please refer to the Data Availability Statement in the manuscript PDF file). The data should be provided as part of the manuscript or its supporting information, or deposited to a public repository. For example, in addition to summary statistics, the data points behind means, medians and variance measures should be available. If there are restrictions on publicly sharing data—e.g. participant privacy or use of data from a third party—those must be specified.  Reviewer #2: No  Reviewer #4: (No Response)  5. Is the manuscript presented in an intelligible fashion and written in standard English?  PLOS ONE does not copyedit accepted manuscripts, so the language in submitted articles must be clear, correct, and unambiguous. Any typographical or grammatical errors should be corrected at revision, so please note any specific errors here.  Reviewer #2: Yes  Reviewer #4: Yes  6. Review Comments to the Author  Please use the space provided to explain your answers to the questions above. You may also include additional comments for the author, including concerns about dual publication, research ethics, or publication ethics. (Please upload your review as an attachment if it exceeds 20,000 characters)  Reviewer #2: The authors are yet to address some of the critical issues raised in the sample size calculation as 2 different sample size were used. Also there are some irregularities observed in the analysis for examples in table 1, the authors did not specify the test of association that was used but only inputted the P value.  Which sampling technique did you used in this study?  Multi-satge technique  Purposive technique at the point of participant recruitment  How relevant is this paragraph to this study and your sample size of 650?  Analysis stated here and did not reflect on the tables in the result section should be deleted.  Kindly re-write this section in a simple and clearer format  Add the name of this flow chart to it eg PRISMA  Which of the test statistics did you do here. Only P-value and no test statistics  Kindly state whether a test of normality was done before Median +/- interquartile range was done  Reviewer #4: (No Response) | The reference list has been reviewed and corrected as advised.  The manuscript has been revised accordingly to remove “see” before “appendix”.  P-value = 0.57 has been added as advised.  A reference (*Hagiwara, Y. and Y. Matsuyama, Goodness-of-fit tests for modified poisson regression possibly producing fitted values exceeding one in binary outcome analysis. Statistical Methods in Medical Research, 2024.* ***33****(7): p. 1185-1196.)* has been added as recommended.  The manuscript has been revised accordingly to remove “see” before all tables and fig. P-values of the adjusted PR have been added in Table 2.  The write up has been edited as advised.  The references have been revised to align with the Vancouver referencing style.  Thanks so much for the feedback.  We appreciate the feedback. We have revised the manuscript extensively.  We appreciate the feedback. We have revised the statistical analysis comprehensively.  For ethical reasons to ensure patient privacy, only anonomyous data presented at group level,  are accessible from the corresponding author on request.  Thanks so much for this feedback.  The critical issues as raised by the reviewer on sample size have been comprehensively addressed in the revised manuscript. The test of association as used in Table 1, have been added as advised.  The manuscript has been revised. The write up now appears as *“We used purposive sampling where participants who met the eligibility criteria and interested in participating in the study, were enrolled consecutively and given their preferred contraceptive choices, until the desired sample size was achieved”.*  We had two objectives in this study;   1. To determine the prevalence of uptake post abortion intrauterine contraception after medical management of first trimester incomplete abortion. 2. To determine the factors associated with the uptake of post abortion intrauterine contraception after medical management of first trimester incomplete abortion.   The relevance of the paragraph is to show the assumptions we used to calculate the sample size for the first objective on the prevalence of uptake of the post abortion intrauterine contraception. We used a study *“Benson J, Andersen K, Healy J, Brahmi D. What factors contribute to postabortion contraceptive uptake by young women? A program evaluation in 10 countries in Asia and sub-Saharan Africa. Global Health: Science and Practice, 2017.* ***5****(4): p. 644-657”*. In this study the prevalence of post abortion IUDs in ten countries in Asia ans Africa, was 11%. Using Open Epi, we got a sample size of 190 participants.  To determine sample size for the second objective on factors associated with the uptake of post abortion intrauterine contraception, we used a study *“Makenzius M, Faxelid E, Gemzell-Danielsson K, Odero TM, Klingberg-Allvin M, Oguttu M. Contraceptive uptake in post abortion care—Secondary outcomes from a randomised controlled trial, Kisumu, Kenya. PloS one, 2018.* ***13****(8): p. e0201214”*. One of the significant factors to the uptake, was the participants’ age. Using Open Epi, we needed a sample size of 642 participants. We compensated for a non-response and had a total sample size of 650 participants. As required, we chose the larger sample size of the two objectives and used this sample to achieve both objectives. It is to this end that we have the sample size of 650 in our study.  The relevance of all these paragraphs, is to enable replicability of study findings in other settings as required of all researchers in regards to the assumptions used to determine our sample size.  Test statistics have been added to the Table 1 as advised. The write up of the analysis section has been revised accordingly.  The name of the flow chart has been edited as advised. It now appears as “*Figure 1: Modified PRISMA Flow diagram of Participants enrolled in the Uptake of Post abortion intrauterine contraception after medical management of first trimester incomplete abortion*  The appropriate test statistics have been added to Table 1 as advised.  A test of normality was done before and when the data was not normally distributed, median and interquartile range were used. The write up now appears as *“Univariate analysis was done using frequencies and percentages for categorical variables and median and interquartile ranges for continuous variables where appropriate. A test of normality was performed to determine whether data was normally distributed or not*. | Page 20-26, line 529-736  Page 6, line 136  Page 10, line 286  Page 10, line 288  Page 14, line 341-343  Page 19, line 495  Page 20-26, line 529-736  Page 7, line 181-184  Page 7-8, line 187-201  Page 11-13, line 316-317  Page 11-13, line 316-317  Page 9, line 246-248 |

Yours truly,

**Dr Herbert Kayiga**

Corresponding Author,

Department of Obstetrics and Gynaecology

Makerere University College of Health Science

+256777855063, [hkayiga@gmail.com](mailto:hkayiga@gmail.com)
