# Supplementary material for: Uptake of intrauterine contraception after medical management of first trimester incomplete abortion: A cross-sectional study in central Uganda
Source: PLoS One. 2025 Oct 7;20(10):e0310936. doi: 10.1371/journal.pone.0310936 (PMC12503278; doi:10.1371/journal.pone.0310936)
Supplement: Attachment — Submitted filename: Revised Response to reviewer comments Uptake.docx [file pone.0310936.s008.docx]

P.O. Box 7072, Kampala, Uganda

Phone: +256-414-534361

e-mail: [obsgyn@chs.mak.ac.ug](mailto:obsgyn@chs.mak.ac.ug)

## COLLEGE OF HEALTH SCIENCES

## SCHOOL OF MEDICINE

## DEPARTMENT OF OBSTETRICS AND GYNAECOLOGY

09^th^ September 2025

RESPONSE TO REVIEWS COMMENTS:

***Object:* PONE-D-24-39454R3** ***“UPTAKE OF INTRAUTERINE CONTRACEPTION AFTER MEDICAL MANAGEMENT OF FIRST TRIMESTER INCOMPLETE ABORTION: A CROSS-SECTIONAL STUDY IN CENTRAL UGANDA”***

With great pleasure, I’m thankful for your comments towards our manuscript. In response to the reviewers’ comments sent to us on 4^th^ September 2025, we have revised the manuscript accordingly.

| **Comment** | **Response to Comment** | **Page No. and Line** |
| --- | --- | --- |
| JOURNAL REQUIREMENTS:  If the reviewer comments include a recommendation to cite specific previously published works, please review and evaluate these publications to determine whether they are relevant and should be cited. There is no requirement to cite these works unless the editor has indicated otherwise.   [Note: HTML markup is below. Please do not edit.]  Reviewers' comments:  Reviewer's Responses to Questions  **Comments to the Author**  1. If the authors have adequately addressed your comments raised in a previous round of review and you feel that this manuscript is now acceptable for publication, you may indicate that here to bypass the “Comments to the Author” section, enter your conflict of interest statement in the “Confidential to Editor” section, and submit your "Accept" recommendation.  Reviewer #2: All comments have been addressed  Reviewer #3: (No Response)  Reviewer #4: All comments have been addressed  Reviewer #5: (No Response)  Reviewer #6: All comments have been addressed  Reviewer #7: All comments have been addressed  2. Is the manuscript technically sound, and do the data support the conclusions?  The manuscript must describe a technically sound piece of scientific research with data that supports the conclusions. Experiments must have been conducted rigorously, with appropriate controls, replication, and sample sizes. The conclusions must be drawn appropriately based on the data presented.  Reviewer #2: Yes  Reviewer #3: Yes  Reviewer #4: Yes  Reviewer #5: No  Reviewer #6: Yes  Reviewer #7: Yes  3. Has the statistical analysis been performed appropriately and rigorously?  Reviewer #2: Yes  Reviewer #3: Yes  Reviewer #4: Yes  Reviewer #5: Yes  Reviewer #6: Yes  Reviewer #7: Yes  4. Have the authors made all data underlying the findings in their manuscript fully available?  The [PLOS Data policy](http://track.editorialmanager.com/CL0/http:%2F%2Fwww.plosone.org%2Fstatic%2Fpolicies.action%23sharing/1/010f019912ed49cd-ea45738c-a264-4420-9ee3-228c2e8d934e-000000/pO586qBs5MB3byL5gIPjxzysK5kYk5nUzUED_e5ga2I=227) requires authors to make all data underlying the findings described in their manuscript fully available without restriction, with rare exception (please refer to the Data Availability Statement in the manuscript PDF file). The data should be provided as part of the manuscript or its supporting information, or deposited to a public repository. For example, in addition to summary statistics, the data points behind means, medians and variance measures should be available. If there are restrictions on publicly sharing data—e.g. participant privacy or use of data from a third party—those must be specified.  Reviewer #2: Yes  Reviewer #3: Yes  Reviewer #4: Yes  Reviewer #5: Yes  Reviewer #6: Yes  Reviewer #7: (No Response)  5. Is the manuscript presented in an intelligible fashion and written in standard English?  PLOS ONE does not copyedit accepted manuscripts, so the language in submitted articles must be clear, correct, and unambiguous. Any typographical or grammatical errors should be corrected at revision, so please note any specific errors here.  Reviewer #2: Yes  Reviewer #3: Yes  Reviewer #4: Yes  Reviewer #5: Yes  Reviewer #6: Yes  Reviewer #7: Yes  6. Review Comments to the Author  Please use the space provided to explain your answers to the questions above. You may also include additional comments for the author, including concerns about dual publication, research ethics, or publication ethics. (Please upload your review as an attachment if it exceeds 20,000 characters)  Reviewer #2: (No Response)  Reviewer #3: I think the writers have done very well with their data analysis and discussion of their results. This is another brilliant article towards advancement of women’s health.  However, some of the concerns raised in the previous review were not addressed although majority of them have been addressed.  Below are some comments that I think need to be attended to as well. This is geared towards making the article better for all.  Reviewers comments  1. Line 98….. two percent among……. and 15% among …….,, I suggest the writer change two percent to 2% or change the 15% to fifteen percent to ensure uniformity. The same must be done for line 311 and all others.  2. Line 141 and 148 are contradictory. In 141, the writers say ultrasound was one of the modalities for dating the pregnancy. But in line 148 they say they only used ultrasound when team suspected incomplete miscarriage or device expulsion. I think they must reconcile the two statements.  3. Lines 150-156 how did the writers obtain consent from the minors who participated in the study(less 18 years ). Did they involve their parents/guardians?    4. Lines 175- 181. should be revised . It’s difficult to understand what the writers are saying. I think the sentence should read, …At the post abortion clinic, potential participants with incomplete abortion were given comprehensive information on either ……..  5. Lines 178-181 “ In Uganda , nurses-midwives are permitted to perform abortion…..” I do not see the relevance of this statement under data collection. It may be appropriate to insert it under introduction.  1. Lines 297-298 should be captured on the table of results  The writers may address the issues raised above.  Reviewer #4: My comments and concerns on the manuscript have been addressed in the first round of review by the authors.  Reviewer #5: This manuscript addresses an important public health issue—determinants of post-abortion intrauterine device (IUD) acceptance. While the topic is timely, the study does not provide sufficiently novel insights beyond what is already known from prior research in similar contexts.  1. The cross-sectional design and the exclusive inclusion of women who already expressed an interest in IUDs introduce severe selection bias. The resulting findings cannot be generalized to the broader population of post-abortion women.  2. No adjustment was made for clustering by facility. Given that counseling practices, provider attitudes, and service availability vary widely across facilities, the lack of multilevel or cluster-adjusted analysis undermines the reliability of the reported associations. 3. Important details of participant recruitment, exclusion, and missing data management are insufficiently described.  4. Safety outcomes and adverse events central to post-abortion contraceptive research are not being reported. Please specify.  5. “Women chose between the Copper IUDs (Nova T®, Bayer AG, Berlin, Germany) and the Levonorgestrel (LNG) IUDs (Mirena®, Bayer AG, Berlin, Germany).” How did these women make their choices? They're not knowledgeable on the subject anyway.  6. “1.9%) participants had exploratory laparotomies following ectopic pregnancies. (Fig 1)” Weren't only incomplete abortion participants included?  Reviewer #6: No further questions or concerns. at this time for the authors. All questions have been answered to the reviewer's satisfaction.  Reviewer #7: (No Response)  7. PLOS authors have the option to publish the peer review history of their article ([what does this mean?](https://track.editorialmanager.com/CL0/https:%2F%2Fjournals.plos.org%2Fplosone%2Fs%2Feditorial-and-peer-review-process%23loc-peer-review-history/1/010f019912ed49cd-ea45738c-a264-4420-9ee3-228c2e8d934e-000000/2Q6A-cykf8Ddj5wCBBeCXbUnVD59jRMfx6UepWo8MnE=227)). If published, this will include your full peer review and any attached files.   If you choose “no”, your identity will remain anonymous but your review may still be made public.   **Do you want your identity to be public for this peer review?** For information about this choice, including consent withdrawal, please see our [Privacy Policy](https://track.editorialmanager.com/CL0/https:%2F%2Fwww.plos.org%2Fprivacy-policy/1/010f019912ed49cd-ea45738c-a264-4420-9ee3-228c2e8d934e-000000/JrVvI-8qoRH_SeYNzYqgFkPgVEIOlKfYSGWsA9Q2rY0=227).  Reviewer #2: No  Reviewer #3: No  Reviewer #4: No  Reviewer #5: No  Reviewer #6: No  Reviewer #7: No  [NOTE: If reviewer comments were submitted as an attachment file, they will be attached to this email and accessible via the submission site. Please log into your account, locate the manuscript record, and check for the action link "View Attachments". If this link does not appear, there are no attachment files.]  While revising your submission, please upload your figure files to the Preflight Analysis and Conversion Engine (PACE) digital diagnostic tool, [https://pacev2.apexcovantage.com/](https://track.editorialmanager.com/CL0/https:%2F%2Fpacev2.apexcovantage.com%2F/1/010f019912ed49cd-ea45738c-a264-4420-9ee3-228c2e8d934e-000000/0p04daJBupqO59ULsz07tsBYfoZ7SY6UvKSUsV_O1jg=227). PACE helps ensure that figures meet PLOS requirements. To use PACE, you must first register as a user. Registration is free. Then, login and navigate to the UPLOAD tab, where you will find detailed instructions on how to use the tool. If you encounter any issues or have any questions when using PACE, please email PLOS at [figures@plos.org](mailto:figures@plos.org). Please note that Supporting Information files do not need this step. | This information is well received.  Thanks so much for this feedback.  Thanks so much for the feedback.  Thanks so much for the feedback.  Thanks for the feedback.  Thanks so much for this feedback.  The manuscript has been revised as advised. The write up now appears as *“Although, intrauterine devices (IUDs) are readily available in Uganda, their user rate has stalled at 2% among the currently married and 15% among the sexually active unmarried women.”*  *Of the other available contraceptive methods, 121(18.6%) women opted for injectable Depo-Provera (DMPA), 116(17.9%) women opted for implants, 35(5.4%) women opted for oral contraceptive pills, 5 (0.8%) of the women opted for condoms, and 3 (0.5%) women opted for periodic abstinence.*  *A third of the participants were aged between (25-29) years. Over 50% of the participants who had abortions, had had a livebirth before. The majority of the participants who had had abortions were living with a partner (84.0%). Forty percent of the participants who had had abortions, had 2-3 children. Only (7)1% of the participants had a monthly income greater than Ugx shillings 1,000,000/= (USD 270). (Table 1)”*    Thanks so much for this observation. We have deleted the aspect of using ultrasound scan in dating of the gestational age. The write up of this section now appears as *“The criteria for identifying study participants with incomplete abortion was based on experience of any of the following conditions: a confirmation of pregnancy by any of the following methods; a positive urine HCG, or calculation from the first day of the last menstrual period. Furthermore, history of lower abdominal pain, and vaginal bleeding, before 12 weeks of gestation was needed to confirm occurrence of first trimester incomplete abortion. Clinical evaluation that included ascertaining cervical dilatation, feeling or visualization of products of conception on vaginal or speculum examination by the clinical team, was conducted to confirm the diagnosis of first trimester incomplete abortion. As indicated in evidence(30), ultrasonography in our study was only used when the clinical team was suspecting incomplete abortion or IUD expulsions.”*  In Uganda minors who conceive are referred to as “emancipated minors” and are allowed to give consent according to the ethical guidelines. In this regard, consent was obtained from minors 15 years or above. Parents/guardians were not involved in the research as permited by the attached ethical approvals from the School of Medicine and Ethics review Committee and the Uganda National Council for Science and Technology.  We appreciate the caution. The intention was to clarify that the participants had to be within 4 weeks after the abortion care. We have however deleted the other aspects. The revised manuscript now appears as *“At the post abortion follow-up visit, potential participants were given comprehensive information on either surgical evacuation (uterine aspiration) or medical evacuation with sublingual misoprostol 400 mcg by trained nurse-midwives.”*  The write up has been deleted from the data collection procedure.  The details as indicated in Lines 297-298 are now reflected in the fig 1 of the revised manuscript.  Thanks so much for this feedback.  We appreciate the feedback.  This study informs policy that even when women express interest in using post abortion intrauterine contraception and all the materials required are fully paid off, nearly 40% of them will opt not to use the method. Though the reviewer might think otherwise, there was a need to explore the factors that influence the uptake of post abortion intrauterine contraception. Since all potential participants were given all the information about all the available contraceptive options, we minimized social desirability bias. With our large sample size of 650 participants from rural, peri urban and urban areas, we are convinced that our findings can be generalized to other areas with similar contextual settings. Our study suggests that further inquiry should be underway to determine the barriers and facilitators of the uptake of post abortion IUDs to minimize on the subsequent unintended pregnancies after first trimester abortions.  Since the study sites had similar context setting, health cadres, all being public facilities offering free family planning services in central Uganda, the relevance of clustering was watered down and the IRB approved an amendment that excluded the clustering effect. The amendment has been attached to this effect.  Though the wording of the subsections might differ, we are convinced that the participant recruitment details are covered under “the participant recruitment section” with further details under “Data collection procedure.” We highlight which patients were identified as potential participants, and the desired patient parameters we sought for. The inclusion and exclusion criteria are indicated. We give an elaborate detail on who the research team members were for the study. We are happy to offer more details as we shall be advised in this regard by the reviewer.  This was a cross sectional study which might have challenges with causal inference to establish cause-and-effect relationships but in the primary study all these aspects are mentioned. We have edited the revised manuscript to add further details of the primary study and the safety and adverse events.  The write up now appears as *“We used a cross-sectional study to determine the level and factors associated with uptake of post abortion IUDs following medical management of first trimester incomplete abortions at five public facilities between 1^st^ February 2023 and 30^th^ September 2023. The participants selected were part of those who were enrolled in the primary study that was non-inferiority open-label randomized controlled study, that compared the expulsion and continuation rates following early insertion (within one week) versus standard insertion (2-4 weeks) after medical management of first trimester incomplete abortion(28). The trial was registered at ClinicalTrials.gov NCT05343546.”*  Both copper and levonorgestrel IUDs are readily accessible in Uganda in nearly 90% of the health facilities. It is to this end that we assumed that the women were knowledgeable of the IUD choices. This was also reinforced by the comprehensive contraceptive counselling services that was offered by the research team prior to enrolment or even utilizing the method.  As requested by one of the reviewers before, a schematic flow of the participants was requested for participants that were included and excluded from the study as indicated in Figure 1. Since the participants had earlier on reported par vaginal bleeding, and lower abdominal pain, though mentioned in text, they were excluded from the study.  Thanks so much for the feedback.  Figure 1 has been edited as per the PACE guidelines. | Page 4, line 95-97  Page 11, line 294-300  Page 6, line 142-150  Page 7, line 177-179  Page 6-7, line 140-186  Page 5, line 110-118  Page 7, line 165 |

Yours truly,

**Dr Herbert Kayiga**

Corresponding Author,

Department of Obstetrics and Gynaecology

Makerere University College of Health Science

+256777855063, [hkayiga@gmail.com](mailto:hkayiga@gmail.com)
